# Entheogen: an evolutionary medicine for neuropsychiatric disorders

**DOI:** 10.1093/nsr/nwaf168

**Published:** 2025-04-25

**Authors:** Yibo Wang, Xiaohui Wang

**Affiliations:** Laboratory of Chemical Biology, Changchun Institute of Applied Chemistry, Chinese Academy of Sciences, China; Laboratory of Chemical Biology, Changchun Institute of Applied Chemistry, Chinese Academy of Sciences, China; School of Applied Chemistry and Engineering, University of Science and Technology of China, China; State Key Laboratory of Brain Machine Intelligence, Zhejiang University, China

Neuropsychiatric disorders represent a significant challenge to modern medicine, encompassing a broad spectrum of conditions that impact both neurological and psychiatric functions of the brain. These disorders include depression, anxiety disorders, obsessive-compulsive disorder (OCD), addiction and post-traumatic stress disorder (PTSD), among others. Given the limited efficacy of existing therapies for these disorders, there is a pressing need for an alternative viewpoint that can provide new insights and lead to more effective intervention strategies.

Viewing neuropsychiatric disorders through the lens of evolutionary medicine offers a compelling perspective, highlighting the intricate interplay among genetic inheritance, environmental changes and evolutionary processes. The ‘mismatch theory’ is central to this understanding, suggesting that many neuropsychiatric disorders stem from a divergence between our ancestral environments and the rapid changes of modern society [1]. One prominent example of this mismatch lies in our altered relationship with nature. In modern urban environments, reduced contact with natural light and settings can negatively impact mental health. While proper light exposure in nature can ameliorate depressive symptoms via specific visual circuits [2], excessive artificial light exposure at night has been linked to increased depressive symptoms, mediated by a neural pathway from retinal melanopsin-expressing ganglion cells to the dorsal perihabenular nucleus (dpHb) and the nucleus accumbens (NAc) [3]. Conversely, consistent studies demonstrate the benefits of spending time in nature, including stress reduction, mood improvement and enhanced cognitive function. Therefore, the absence of regular exposure to nature, a constant in our ancestral past, likely contributes to mental health challenges in contemporary urban settings. Similarly, modern social isolation contrasts sharply with the tightly knit social structures of our ancestors, which provided crucial buffers against stress. This social mismatch can further exacerbate neuropsychiatric vulnerabilities. Evolutionary medicine also highlights the role of natural products in therapeutic development. The concept of animal self-medication, or zoopharmacognosy, suggests an evolutionary adaptation where species, including humans, instinctively utilize specific plants or substances for disease treatment and prevention. This behavior reflects an inherent drive towards healing and maintaining homeostasis. Humans have long leveraged plant-based and organism-derived remedies, tapping into evolved chemical defenses for medicinal purposes. This highlights the importance of exploring biodiversity, a natural pharmacopeia refined over millions of years, as a source of novel therapeutic agents. For example, entheogens, psychoactive substances derived from plants (e.g. mescaline in peyote), fungi (e.g. psilocybin in magic mushrooms) and animals (e.g. bufotenin in some species of toads), have been utilized for millennia in religious, shamanic or spiritual contexts to elicit altered states of consciousness aimed at increasing self-acceptance, healing, or gaining insights. While often associated with religious practices, we use ‘entheogen’ to encompass psychoactive substances capable of inducing profound altered states of consciousness, regardless of context. From psychedelics like psilocybin and ayahuasca to other psychoactive substances, these compounds have been part of human cultures for millennia, suggesting an evolutionary aspect to their use. The altered states induced by entheogens may facilitate a reconnection with nature and enhance social connection and empathy, potentially mitigating the negative effects of modern environmental mismatches.

From an evolutionary medicine viewpoint, entheogen use can address the dissonance between our ancient biology and modern neuropsychiatric challenges. In ancestral societies, ritualistic entheogen uses likely played a crucial role in community bonding, stress alleviation and perhaps even the facilitation of neuroplastic changes that could aid in coping with trauma or psychological stressors, contributing to social cohesion and individual mental health. Given the prevalence of brutal killings and wars throughout human history, the role of entheogens assumes particular significance. For instance, the Native Americans of the New World, who endured centuries of slaughter, likely found psychedelics to be crucial in healing their collective psychological wounds, highlighting the enduring importance of entheogens in navigating the challenges posed by societal upheavals and conflicts.

Modern research reveals the therapeutic potential of entheogens for conditions such as depression, PTSD and anxiety, often exceeding the benefits of conventional medications, sometimes with lasting effects from a single administration. For instance, a 2024 systematic review and meta-analysis of nine studies (436 participants) reported a significant benefit from psilocybin (Hedges’ g = 0.66, 95% confidence interval (CI) 0.46 to 0.86, *P* < 0.001), indicating a medium to large effect on depression symptoms [4]. The likelihood of achieving a treatment response with psilocybin was approximately twice that of placebo (risk ratio 2.17, 95% CI 1.18 to 3.99), while the chance of achieving remission from depression was nearly three times greater (risk ratio 2.96, 95% CI 1.69 to 5.17). Additionally, subgroup analyses showed larger effects for secondary depression and self-report depression scales, with metaregression indicating that older age and prior psychedelic use were associated with greater improvement. However, careful consideration of different doses and treatment regimens is essential. For instance, a single 25 mg dose of psilocybin, but not a 10 mg dose, significantly reduced depression scores compared to a 1 mg dose over a period of three weeks, suggesting dose-dependent efficacy [5]. Furthermore, a 2023 dose-response meta-analysis found varying 95% effective doses per day: 8.92 mg/70 kg for secondary depression, 24.68 mg/70 kg for primary depression and 40 mg/70 kg for treatment-resistant depression, indicating higher doses are needed for more severe cases and treatment regimens might benefit from dose personalization [6].

These substances enhance neural plasticity and promote connectivity within the brain, potentially enabling patients to overcome rigid, negative thought patterns. Classically, psychedelics like psilocybin and LSD act as 5-HT_2A_ receptor agonists, inducing widespread alterations in neuronal activity. This receptor activation disrupts typical neural communication patterns, increasing neural entropy and modulating brain network activity, initiating increased expression of brain-derived neurotrophic factor (BDNF), a key protein involved in neuronal growth, survival and synaptic plasticity. This suggests a mechanism by which these substances may promote the formation of new neural connections and strengthen existing ones. Neuroimaging studies using fMRI have demonstrated that psychedelics significantly modulate the activity of the default mode network (DMN), a brain network associated with self-referential thought and rumination [7]. This modulation may facilitate the disruption of rigid thought patterns and habitual modes of thinking characteristic of conditions like depression and anxiety. Furthermore, psychedelics may also enhance activity in other brain regions associated with emotion processing and social cognition.

This ability of entheogens to ‘reboot’ the brain by resetting maladaptive neural pathways aligns with the evolutionary perspective that, although our brains have developed to be highly adaptable and responsive to environmental changes, they may still manifest maladaptive responses in contexts that differ significantly from those of our ancestors. Thus, entheogens act as a bridge across this evolutionary gap, offering therapeutic interventions that draw upon our ancient past to meet the mental health challenges of today. This therapeutic impact of psychedelics derives partly from their ancient role in increasing prosocial behavior and enhancing empathy, suggesting a deep-rooted biological predisposition towards their beneficial effects on mental health. Recognizing this potential, psychedelics have received significant regulatory acknowledgment, with four FDA Breakthrough Therapy designations awarded to substances like psilocybin for depression and anorexia nervosa, MDMA (Ecstasy) for PTSD, and LSD for anxiety. This FDA recognition highlights a growing acceptance and interest in leveraging the therapeutic benefits of psychedelics to tackle complex neuropsychiatric conditions effectively. Indeed, Australia has made significant strides in the recognition and approval of psychedelics for therapeutic uses. The country has approved psilocybin, for the treatment of depression, and MDMA, for PTSD, since 1 July 2023. This groundbreaking decision marks a pivotal shift in the medical and regulatory landscape, acknowledging the potential of these substances to offer relief where traditional treatments may fall short.

The communal and ritualistic use of entheogens in traditional societies highlights the significance of social and environmental contexts in healing, a dimension often neglected in modern psychiatric treatments. These historical practices, rooted in rituals aimed at community bonding, stress alleviation and psychological trauma management, have fostered social cohesion and well-being. Such practices emphasize the profound impact of ‘set’, the individual's psychological state, including expectations, mood and emotions, and ‘setting’, the physical and social environment of consumption, encompassing surroundings, company, comfort and safety, on the psychedelic experience [8]. The interplay between set and setting significantly impacts improvements in mood, anxiety and overall psychological well-being, along with increased self-efficacy and adaptive coping strategies. For instance, ayahuasca used in Amazonian shamanic ceremonies, led by experienced shamans, provides a ritualistic context that enhances the experience and strengthens community bonds. Modern psychedelic research and therapy, recognizing the value of these traditional practices, emphasizes meticulous preparation and safe therapeutic environments to ensure positive outcomes. By integrating insights from evolutionary medicine with the wisdom of traditional entheogen use, contemporary approaches can draw upon this rich tapestry of knowledge to enhance modern psychedelic treatments, fostering a more holistic approach to mental health that integrates social, spiritual and biochemical dimensions of healing.

While modern therapies draw inspiration from traditional practices, their contexts and goals differ. Traditional use is often embedded in religious rituals and spiritual beliefs, aiming for spiritual healing and community bonding. Modern clinical applications operate within scientific and ethical frameworks, focusing on specific mental health conditions using standardized protocols. Despite these differences, key principles like set, setting, preparation and integration have been integrated into modern therapy. Both approaches recognize the therapeutic potential of altered states of consciousness. However, integration presents challenges. Modern applications adhere to strict ethical standards and employ trained therapists focused on psychological processing. Nonetheless, this integration of traditional wisdom and modern science offers significant potential for holistic psychedelic therapies.

While psychedelics offer therapeutic promise, associated risks must be addressed. These substances can induce potential adverse reactions, including acute psychological effects like anxiety, fear or confusion, often referred to as ‘bad trips’, and physical effects such as increased heart rate, blood pressure, nausea and vomiting. Concerns also exist regarding potential long-term psychological effects, such as hallucinogen persisting perception disorder, psychotic symptoms, anxiety and fear among certain populations, or the exacerbation of pre-existing mental health conditions, especially in individuals with a history of psychosis or bipolar disorder. Although these long-term effects are rare in controlled clinical settings, they warrant careful consideration. For instance, research on psilocybin for depression indicates that common adverse effects in clinical trials include headache, nausea and anxiety, but no serious adverse events have been reported [9]. These findings suggest that while risks are present, they are generally mild or moderate in severity, transient and expected. However, a detailed analysis is essential to inform clinical practice, particularly given the limited evidence regarding who might be affected and under what circumstances. While research demonstrates promising short-term effects, long-term impacts and dependence potential require consideration. Evidence suggests that classic psychedelics like LSD and psilocybin generally do not cause long-term cognitive impairment in individuals without pre-existing vulnerabilities when used in controlled settings [10,11]. However, further research is needed, especially regarding repeated use. Although physical dependence is unlikely, psychological dependence or misuse (use inconsistent with therapeutic guidelines) remains a concern, particularly in uncontrolled settings or among vulnerable individuals. It is important to distinguish misuse from dependence: dependence involves compulsive use despite negative consequences, while misuse may not.

As entheogen-based therapies advance toward medical approval and extramedical use rises, strategies can be applied to effectively manage the adverse effects in clinical practice. First, patients should be screened for contraindications, such as a history of psychotic disorders, severe cardiovascular disease, or other conditions that might be exacerbated by entheogen use. This involves comprehensive psychiatric and medical evaluations to identify individuals at higher risk for adverse reactions. Second, therapy sessions should be conducted in a safe, comfortable environment with skilled mental health professionals present to monitor and guide the patient. This setting, often referred to as ‘set and setting’, is crucial for minimizing risks. A supportive environment reduces the likelihood of challenging experiences, with therapists trained to provide psychological support during the session. Third, patients should undergo preparation sessions to understand the treatment process, set intentions and learn coping techniques for managing potential distress. Post-session integration sessions help patients process their experiences, integrate insights and address any challenging aspects, reducing the risk of lingering psychological effects. Studies have emphasized the role of integration in enhancing therapeutic outcomes and managing risks [12]. During sessions, patients should be continuously monitored for vital signs (e.g. heart rate, blood pressure) and psychological state. Protocols should be in place to manage any acute distress or medical emergencies, such as providing a calm, grounding environment or, in rare cases, administering anxiolytics if necessary. Lastly, regular follow-up sessions are essential to address any ongoing effects or issues and to support the patient's integration process. This includes monitoring for any delayed psychological effects and providing additional therapy if needed. Long-term follow-up data from trials show that with proper follow-up, the incidence of adverse effects is minimal [13].

While the potential adverse effects of entheogens can be significantly reduced through rigorous patient selection, controlled settings, preparation/integration, monitoring and follow-up care, another promising avenue lies in directly modifying these compounds to eliminate their hallucinogenic and other undesirable side effects. Compounds such as tabernanthalog (TBG), a psychedelic analog, have shown potential for anti-addictive and antidepressant effects, while exhibiting fewer side effects compared to traditional psychedelics [14]. This approach not only improves safety but also broadens clinical applicability, particularly for populations at risk for cardiovascular issues or other concerns related to side effects. In addition to pharmacological strategies, we have recently proposed a non-invasive modulation strategy using Terahertz waves [15]. This innovative approach leverages Terahertz waves with specific frequencies to selectively enhance entheogen interactions with therapeutic receptors while reducing their affinity for receptors associated with adverse effects. This method holds the potential to further increase the clinical adoption of entheogens by maximizing therapeutic benefits while minimizing side effects. Collectively, these studies illustrate how evolutionary insights into entheogen use, combined with modern scientific advancements, can guide the design of safer, more effective treatments for mental disorders, ultimately modernizing ancient practices with cutting-edge technology.

In conclusion, exploring entheogens through an evolutionary lens provides a persuasive argument for their use in treating neuropsychiatric disorders. This viewpoint suggests that historical entheogen use offered evolutionary benefits in managing psychological stress, informing contemporary therapeutic strategies. This aligns treatment with our evolutionary past, promoting a comprehensive approach to mental health that honors our biological and evolutionary intricacies. By integrating evolutionary insights with the pursuit of novel treatments, we are poised for a paradigm shift in addressing neuropsychiatric conditions. Techniques inspired by ancestral practices, such as mindfulness and meditation, highlight the potential for effective interventions resonant with our evolutionary heritage. Thus, entheogens, viewed through this lens, offer a promising path forward, advocating for an innovative treatment model deeply rooted in our collective past.
